# Supplementary material for: Cuticular profiling of insecticide resistant Aedes aegypti
Source: bioRxiv. 2023 Jan 13:2023.01.13.523989. Preprint. [Version 1] doi: 10.1101/2023.01.13.523989 (PMC9882251; doi:10.1101/2023.01.13.523989)
Supplement: 1 [file NIHPP2023.01.13.523989v1-supplement-1.pdf]

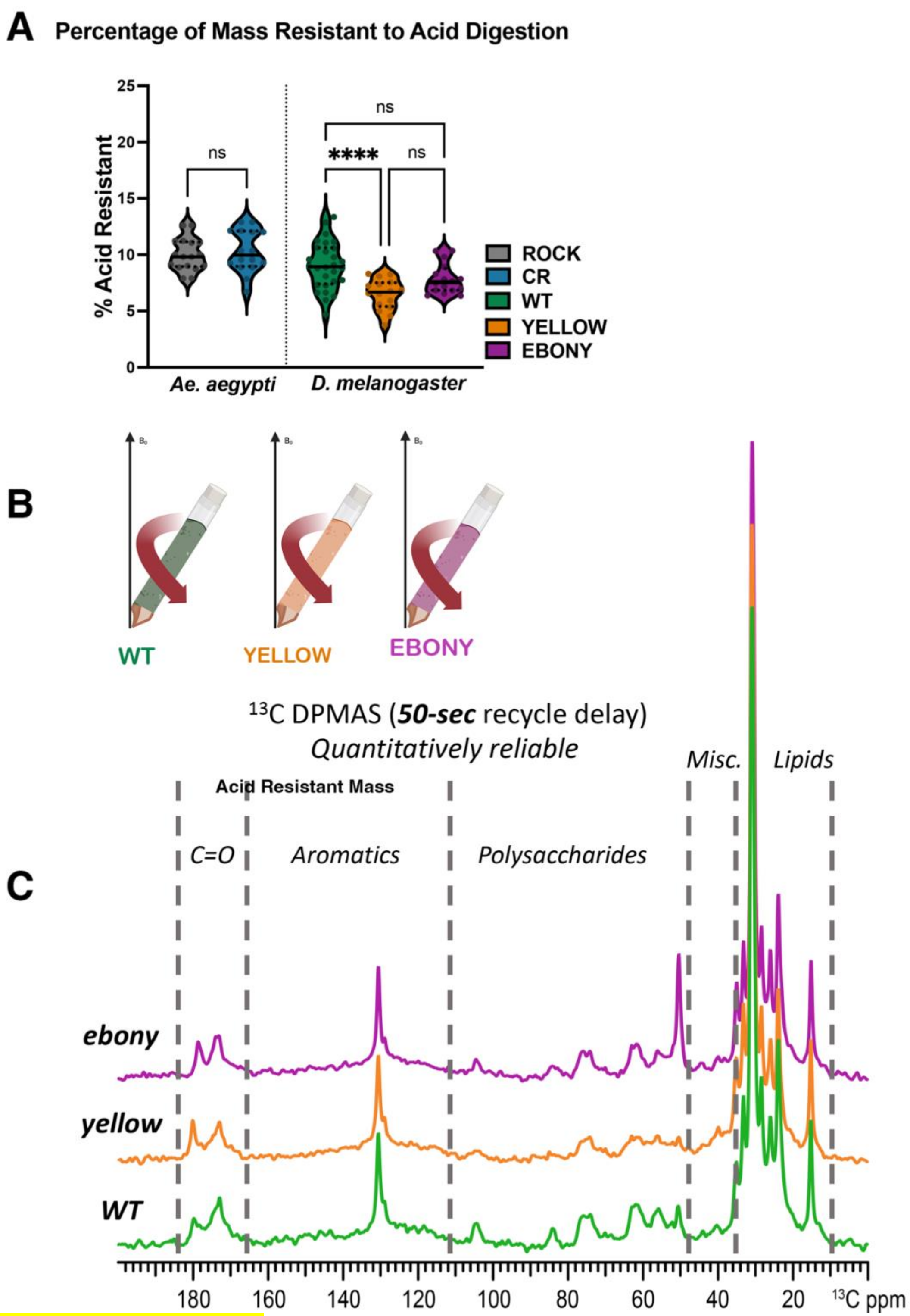

Supplementary Figure 1

A: Percentage of female *Ae. aegypti* ROCK (grey) and CR (dark blue) and *D. melanogaster* WT (green), yellow (orange), and ebony (pink) wet weights that were resistant to acid digestion. All digestion samples contained 25 females each across three pooled biological replicates. Sample number: CR n = 16, ROCK n = 17, WT n= 28, yellow n= 16, Ebony n = 16. One-way ANOVA with

Tukey's Multiple Comparison test p value: ****= <0.0001, ** = 0.0048 **B:** Schematic of material loaded into ssNMR rotor to compare acid-resistant material from *D. melanogaster* strains **C:** direct-polarization (DPMAS) Carbon-13 (^{13}C) ssNMR (50-sec delay; quantitatively reliable) comparison of acid-resistant material of the WT (green), yellow (orange), and ebony (pink) strains pooled from three biological replicates.

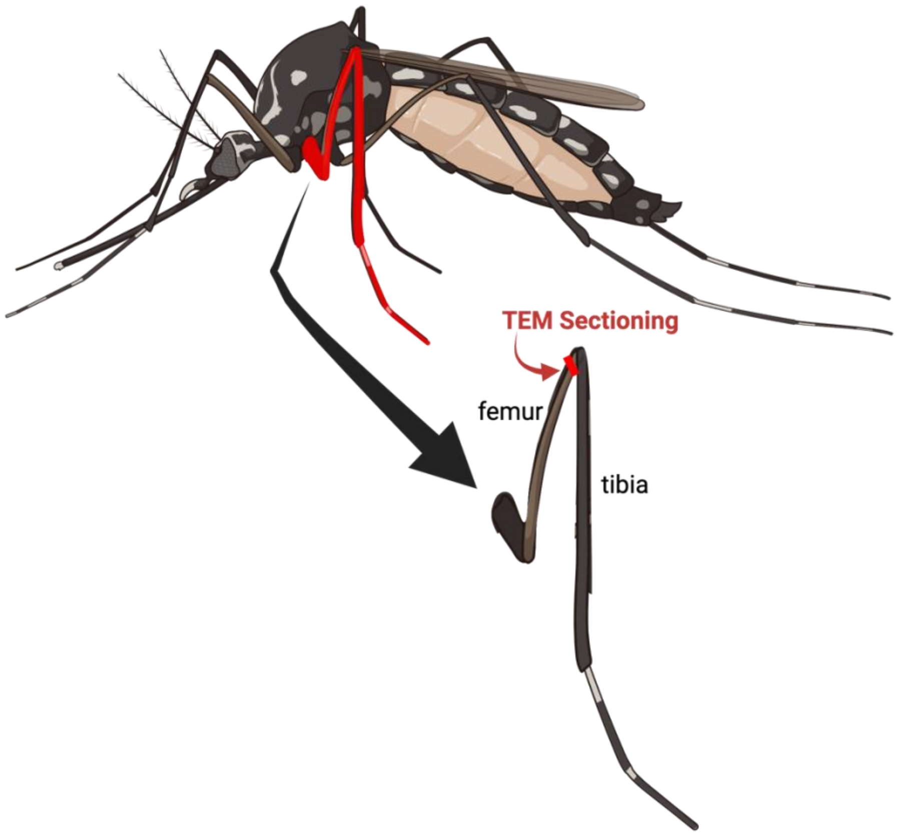

Supplementary Figure 2

A: Schematic of TEM sectioning performed 200 nm into the midleg femur.
